# Alkali treatment–acid leaching of rare earth elements from phosphogypsum fertilizer: insight for additional resource of valuable components

**DOI:** 10.1186/s13065-022-00845-7

**Published:** 2022-07-09

**Authors:** M. S. Gasser, Z. H. Ismail, E. M. Abu Elgoud, F. Abdel Hai, I. O. Ali, H. F. Aly

**Affiliations:** 1 Hot Laboratories Center, Egyptian Atomic Energy Authority, Cairo, 13759 Egypt; 2 Chemistry Department, Faculty of Science, Al-Azhar University, Cairo, Egypt

**Keywords:** REEs, Phosphogypsum fertilizer, Alkali-acid, Leaching

## Abstract

Phosphogypsum (PG) is the main by-product of phosphoric acid, which is produced by the sulfuric acid attack of phosphate rocks, wet process. This by-product, which contains around 2.0% phosphoric acid, is used as a low-cost soil fertilizer, PGF. PGF consists mainly of gypsum (CaSO_4_·2H_2_O), P_2_O_5_, SiO_2,_ and other impurities, including a minor amount of rare earth elements, REEs. In general, phosphate rocks contain from about 0.04 to 1.0% REE, which are precipitated with PG. Now, REEs are considered as strategic elements. Therefore, PG is now regarded as a secondary source of REE. This paper address a process for the separation of REEs and sodium sulphate as a product from PGF. This paper is based on the metathesis of the bulk of PGF with sodium carbonate to obtain calcium carbonate precipitated contain REEs. Furthermore, sodium sulphate was obtained as a product. Calcium carbonate containing REEs was leached out by citric acid as a green acid or nitric acid. At optimum conditions, maximum leaching of REEs from CaCO_3_ after one cycle of leaching by 3.0 mol/L nitric acid at L/S = 3/1, agitation time of 180.0 min., and at a temperature of 25 °C is 75.1%, 361.10 mg/kg from the total REEs present in PGF. While, the maximum leaching of 87.4%, 420.2 mg/kg of REEs from CaCO_3_ after one cycle of leaching by 1.0 mol/L citric acid, L/S = 5/1, agitation time of 15.0 min., and 85 °C. The REEs that were obtained in the leaching citrate solutions were purified by solvent extraction using 10% of di-2-ethyl hexyl phosphoric acid, HDEHP, in kerosene. The extracted REEs were stripped by 0.5 mol/L H_2_SO_4_. The stripped solutions were further treated with 10.0% oxalic acid to precipitate the REEs. The developed procedure can recover REEs from PGF with an efficiency of 85.2% and a purity of 97.7%.

## Introduction

Phosphogypsum (PG) is a byproduct generated during the industrial wet process of phosphoric acid production, in which sulfuric acid is used to digest phosphate rock. Gypsum (CaSO_4_·2H_2_O), the main component of PG, usually accounts for 65.0 to 95.0% of PG by weight. There are small quantities of impurities in PG, such as phosphates (H_3_PO_4_, Ca(H_2_PO_4_)_2_.H_2_O, CaHPO_4_.2H_2_O, and Ca_3_(PO_4_)_2_), fluorides (NaF, Na_2_SiF_6_, Na_3_AlF_6_, Na_3_FeF_6_, and CaF), sulfates, trace metals, and radioactive elements [1]. The large-scale production of these undesirable by-products, i.e., over 100–280 Mt/yr of PG worldwide [2, 3], but only about 15.0 percent, were reused as building materials, agricultural fertilizers, or soil stabilization amendments [4]. The remaining 85% are considered wastes that require large disposal areas and may cause huge environmental problems because of the high content of metals and impurities [5, 6]. Therefore, most common waste treatment practices have traditionally concentrated on relieving the release of contaminants by covering PG piles with impermeable materials and collecting acid effluents for further treatment. On the other hand, PG is regarded as an important REEs secondary resource. The waste typically contains 0.04 to 1.0% of REEs. These elements are critical materials for green energy development due to their essential roles in items like lamp phosphors and permanent magnets, catalysts, and rechargeable batteries [7, 8]. Although research has been conducted, a technology that allows the developer to economically recover these REE elements from the PG waste has not yet been developed [9–16]. Furthermore, the existence of radioactivity overwhelmingly restricts PG utilization. In the United States, the use of PG was banned in 1990 [17], and in the European Union, it was discontinued in 1992 because of the potential radiological impact.

Research-based on hydrometallurgical focused on methods of recovering REEs in PG [18, 19]. The recovery of REEs could be considered a promising, economic, and environmentally friendly solution for the management of these wastes. However, the huge volume of PG landfilled near fertilizer industries may contain enough REEs to be mined if selective retrieval methods are advanced [13, 14, 20–25]. Lütke et al. [26] investigated the leaching of rare earth elements from PG by using citric and sulfuric acid. They reported that the leaching efficiency values of total rare earth elements were 62.0% and 89.7% for citric and sulfuric acid, respectively. Cánovas et al. [27] studied the leaching of REEs from PG with nitric and sulfuric acid. The obtained results indicated that the high leaching efficiency of REEs above 80.0% was achieved by using 3.0 mol/L nitric acid. While the leaching efficiency by using 0.50 mol/L sulfuric acid is in the range of 46.0–58.0%. Ennaciri et al. [28] developed a process for the production of K_2_SO_4_ by the conversion of phosphogypsum (CaSO_4_. 2H_2_O) and potassium carbonate (K_2_CO_3_). The obtained result showed that the reaction was conducted with stoichiometric ratios between PG and potassium carbonate and the high conversion of PG was achieved at 80 ^◦^C. Production of rare earth elements from PG after treatment with sodium chloride followed by sodium carbonate has been studied by Hammas-Nasri et al. [29]. They found that the total rare earth enrichment of about 84% was achieved in the final solid by using a washing step with (25 g/L) NaCl followed by leaching the residue with (60 g/L) Na_2_CO_3_ at 90 °C for 60.0 min. Leaching of rare earth elements from PG using different mineral acids (HCl, H_2_SO_4,_ and HNO_3_) has been examined by Walawalkar et al. [30]. They reported the leaching efficiency of REEs was 57.0%, 51.0%, and 32.0% for HNO_3_, HCl, and H_2_SO_4_, respectively. Hammas-Nasri et al. [31] employed dilute sulfuric acid for the leaching of REEs from PG waste by a two-step leaching method. Their work showed that the leaching efficiency of REEs was about 50.0% by using double leaching with a 10.0% sulfuric acid solution at 60 °C for 1.0–2.0 h and a liquid/solid ratio of 1.3. Guan et al. [32] evaluated the behavior of hydrochloric acid for the leaching of REEs from PG. The experimental results showed that the maximum leaching efficiency for REEs was 65.6% at operating conditions (acid concentration of 1.65 mol/L, S/L ratio of 1/10, and reaction temperature of 60 °C).

Recently, we developed a process for REEs with citric acid as a green acid, by direct leaching of PGF and PG containing 2.0% H_3_PO_4_ with a leaching efficiency of more than 84.0%. [13] In this work, this process was modified to enable efficient recovery of REEs, which were purified and separated. In this concern, a metathesis reaction based on the transformation of the precipitated calcium sulfate-free from REEs in the PGF to calcium carbonate precipitate containing the REEs by sodium carbonate with the release of the sodium sulfate into the solution. Further, the REEs precipitated with calcium carbonate were leached out by the use of a green citric and nitric acid solution and further purified by solvent extraction.

## Experimental

### Chemicals and reagents

All chemicals used were of analytical grade unless stated otherwise. Citric acid, AR, was supplied from Adwic, Egypt. Different REEs, AR, were obtained as oxides from Fluka. The extractant HDEHP was purchased from Aldrich. The odorless kerosene was used as a diluent for the extractant and obtained from Misr Petroleum Company, Egypt.

## PGF characteristics

PGF samples were obtained from Abu-Zaabal Fertilizers Company and Chemicals, Egypt. In the previous work [14], PGF was characterized using X-ray fluorescence spectrometry, XRF, X-ray diffraction, XRD, Infrared spectrum spectroscopy, FT-IR, and an Inductively Coupled Plasma Optical Emission Spectrometer (ICP-OES). In this concern, the major elemental chemical analysis of PGF, which was done by XRF, was given in Table 1. The total REEs in the PGF sample was equal to 481.0 ± 5 mg/kg, Table 2.

**Table 1:** Chemical analysis of PGF by X- ray fluorescence (XRF)

Analyte	Compound formula	Conc. (%)
F	F	0.36
Na	Na_2_O	0.24
Mg	MgO	0.24
Al	Al_2_O_3_	0.26
Si	SiO_2_	9.95
P	P_2_O_5_	2.38
S	SO_3_	44.08
Ca	CaO	35.9
Sr	SrO	0.16
Ti	TiO_2_	0.15
Fe	Fe_2_O_3_	1.64
Ni	NiO	0.12

**Table 2:** Chemical analysis of REEs in PGF by ICP–OES

Element	Concentration (mg/kg)
La	117.0
Ce	234.1
Pr	27.1
Nd	< 0.1
Sm	2.0
Er	79.1
Y	21.6
Yb	< 0.1
Total REEs	~ 481.0 mg/kg

**Fig. 1:**
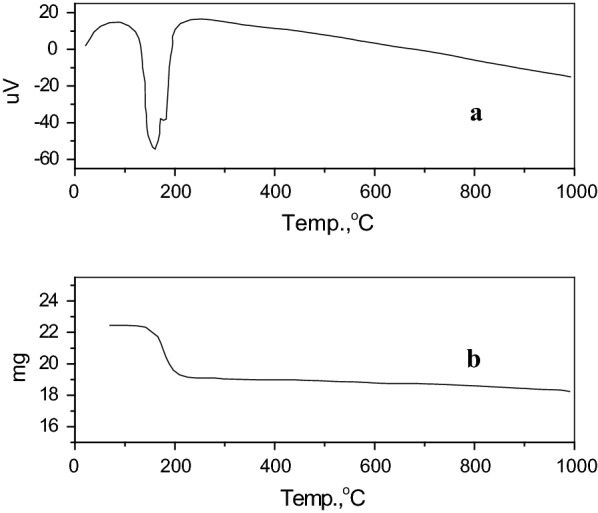
Thermal analysis **a** DTA **b** TGA of PGF

Thermal analysis, differential thermal analysis (DTA), and thermal gravimetric analysis (TGA) were performed on a Shimadzu DTG–60/60 H with a heating rate of 20 °C/min under N_2_ flow. The differential thermal analysis (DTA) of the PGF sample shows the presence of two endothermic peaks, Fig. 1a. The first one occurred at 160.5 °C and may be related to a loss of 1.5 mol of H_2_O from dihydrate calcium sulphate (CaSO_4_.2H_2_O) and the formation of hemihydrate calcium sulphate (CaSO_4_.1/2 H_2_O) according to Eq.  [33]:

1 $${\text{CaSO}}_{4} \cdot {\text{2H}}_{{2}} {\text{O}}\,\mathop{\longrightarrow}\limits^{{\Delta , \sim \,160.5\,^\circ {\text{C}}}}\,{\text{CaSO}}_{4} \cdot {\raise0.5ex\hbox{$\scriptstyle 1$} \kern-0.1em/\kern-0.15em \lower0.25ex\hbox{$\scriptstyle 2$}}{\text{H}}_{2} {\text{O}} + {\raise0.5ex\hbox{$\scriptstyle 3$} \kern-0.1em/\kern-0.15em \lower0.25ex\hbox{$\scriptstyle 2$}}{\text{H}}_{2} {\text{O}}$$

The TGA curve, Fig. 1b, of the PGF sample, shows a weight loss of 15%. Part of this weight loss may be due to humidity, and the other part corresponds to the endothermic DTA peaks. It was observed that the phase transition of hydrated calcium sulfate (CaSO_4_.2H_2_O) in PG to hemihydrate and un-hydrated calcium sulfate using DTA and TGA analysis [33, 34]. These two endothermic peaks appeared at 151 °C and 180 °C while the weight loss was 18.2%. The shift in peaks and the difference in weight loss may be attributed to the purity of PGF, the amount of residual acid present, and the origin of phosphate rock used for phosphoric acid production.

### Leaching investigation

#### Sample preparation

The PGF sample was dried at 200 °C for 2 h and then analyzed. Chemical analysis of the dried sample was shown in Table 3, which indicated that there was no change in the chemical composition of PGF due to heating.

**Table 3:** Chemical analysis of PGF by XRF, after drying

Analyte	Compound formula	Conc. %
F	F	0.33
Na	Na_2_O	0.30
Mg	MgO	0.26
Al	Al_2_O_3_	0.26
Si	SiO_2_	10.06
P	P_2_O_5_	2.52
S	SO_3_	46.22
Ca	CaO	39.38
Sr	SrO	0.27
Ti	TiO_2_	0.19
Fe	Fe_2_O_3_	1.54
Ni	NiO	0.25

#### Leaching process

Unless otherwise stated, leaching experiments were held by taking a certain known volume of the leaching solution in a polyethylene vial with 1.0 g of PGF and mixing thoroughly for a predetermined period. The admixture is separated by filtration and the total concentration of the resulted REEs (mg/L) is specified in the leaching solution calorimetrically by the Arsenazo-III method [35]. The Shimadzu UV–visible spectrophotometer model UV-160, Japan, was used to measure the concentrations of total REEs in samples after investigation. Individual REEs were determined by ICP-OES.

Dried PGF sample was mixed with a certain volume of 0.4 mol/L Na_2_CO_3_ for 120.0 min. at 25 °C, the formed mixture was filtrated and the solid residue was treated with nitric acid or citric acid. In this concern, 3.0 mol/L of nitric acid was used with L/S ratio of 3/1 at a temperature of 25 °C and a contact time of 180.0 min. While 1.0 mol/L citric acid was used at L/S ratio of 5/1 at a temperature of 85 °C and a contact time of 15.0 min.

The total percent of REEs leached (total% of REEs leached) was calculated using the Eq. (2);

2 $${\text{Total}}\,{\text{percentage}}\,{\text{of}}\,{\text{REEs}}\,{\text{leached}}\, = \left[ {{\text{C}}_{f} /{\text{C}}_{o} } \right] \times \,100$$

where, C_o_ is the concentration of the total REEs (mg/L) actually present in 1.0 g of PGF. To determine C_o_, 1.0 g of PGF was completely dissolved in aqua regia and evaporated until dryness. [13]

### Extraction procedure

Leaching of REEs from PGF with 1.0 mol/L citric acid at an L/S ratio of 5/1, a temperature of 85 °C, and equilibrium time of 15.0 min was carried out. The obtained leaching solution was contacted with an equal volume of organic solution with a known HDEHP concentration in kerosene. The two phases were shaken for a predetermined period in a thermostated mechanical shaker. After equilibration, the two phases are separated using a separating funnel. The REEs concentration extracted in the organic phase was calculated by the difference between its concentration in the aqueous phase before and after extraction.

## Results and discussion

In the previous work, the leaching behavior of the total lanthanides, REEs, from PGF has been examined using nitric acid, hydrochloric acid, and sulfuric acid [14]. Recovery was highest when the PGF was leached with 3.0 mol/L HNO_3_. In the last work, some organic acids, namely boric acid, malic acid, and citric acid were used to leach REEs (Ln-Y) from PGF [13]. It was concluded that the 1.0 mol/L citric acid solution was the most effective leaching solution for REEs from PGF compared to other acids.

Based on the aforementioned results, the combined process for leaching the REEs from PGF was developed. The process was based on the alkaline dissolution of the PGF by sodium carbonate solution to form soluble sodium sulphate as product and a precipitate of calcium carbonate together with the different REEs. This is followed by one-cycle leaching of the REEs from the carbonate precipitate with either nitric acid or citric acid. In this respect, the PGF sample was treatment with sodium carbonate (0.40 mol/L) [36] for 120.0 min at 25 °C to produce sodium sulphate according to the following equation:

3 $${\text{CaSO}}_{{4}} \, + \,{\text{Na}}_{{2}} {\text{CO}}_{{3}} \, \to \,{\text{Na}}_{{2}} {\text{SO}}_{{4}} \, + \,{\text{CaCO}}_{{3}} \, \downarrow$$

There are several uses for sodium sulphate as a filler in powdered home laundry detergents and other uses. REEs are associated with CaCO_3_ and an analysis of the total REEs present in sodium sulphate solution was found to be less than 1.0% as indicated in Table 4.

**Table 4:** The concentration of REEs present in a sodium sulfate solution

Element	Conc., (mg/kg)
La	1.3
Ce	3.7
Pr	–
Yb	–
Sm	–
Er	–
Y	–
Total REEs	~ 5.021 mg/kg ~ 1%

[Na_2_CO_3_] = 0.40 mol/L agitation time = 120.0 min.

T = 25 °C

### Alkali treatment-nitric acid leaching

**Fig. 2:**
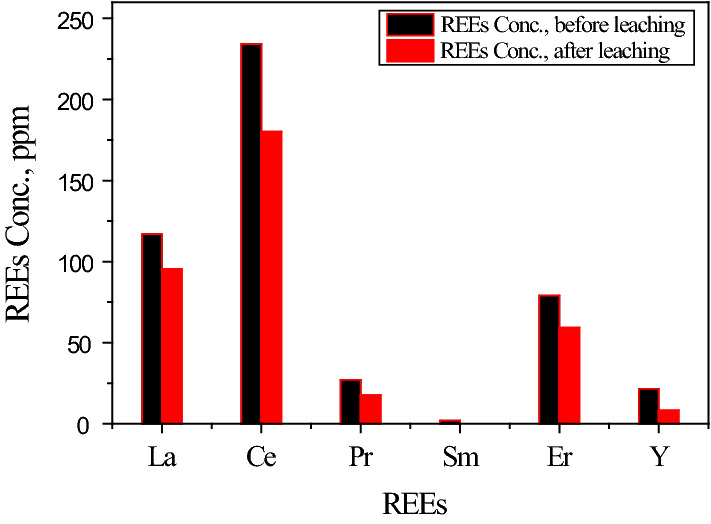
Concentrations of different REEs, ppm in PGF, and that leached from one-cycle. with 3.0 mol/L nitric acid solution, at agitation time 180.0 min, L:S = 3:1 and 25 °C.

In previous work [14], nitric acid was used to leach REEs directly from PGF by three-cycle. The maximum leaching efficiency of REEs was 66.0% using 3.0 mol/L nitric acid. Nevertheless, when nitric acid was used to leach the calcium carbonate containing REEs in the present work was found higher than 75.0% under a similar condition. After filtration, the obtained leaching solution was analyzed as illustrated in Fig. 2. From this figure, it is clear that the total REEs obtained in one cycle of leaching by nitric acid are 75.1% of the total REEs present in PGF.

### Alkali treatment- citric acid leaching

**Fig. 3:**
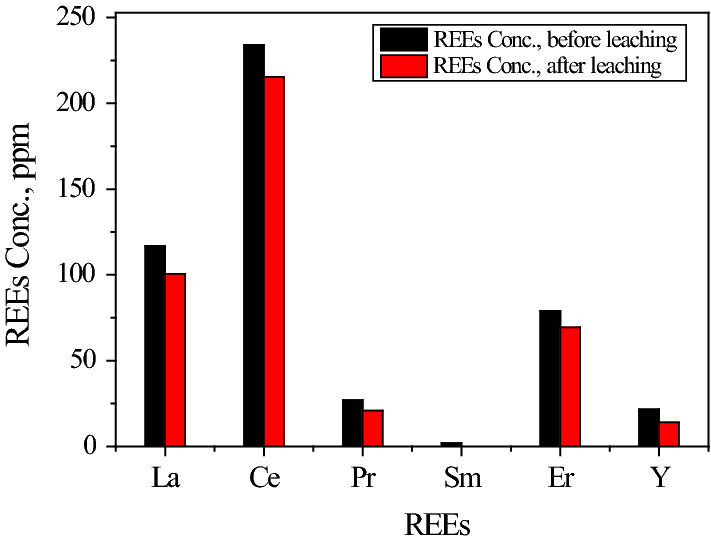
Concentrations of different REEs, ppm in PGF, and that leached from one-cycle with 1.0 mol/L citric acid solution, at agitation time 15.0 min, L:S = 5:1 and 85 °C.

In previous work [13], citric acid was utilized to leach REEs directly from PGF by three-cycle. The maximum leaching efficiency of REEs was 83.4% using 1.0 mol/L citric acid. Moreover, when citric acid was used to leach the calcium carbonate containing REEs in the present work was found higher than 87.0% under a similar condition. The optimum conditions, 1.0 mol/L citric acid, an L/S ratio of 5/1, equilibrium time of 15.0 min at 85 °C. After filtration, the obtained leaching solution was analyzed as given in Fig. 3. From this figure, it is clear that the total REEs obtained from one cycle of leaching by citric acid are 87.4% of the total REEs present in PGF.

The residue that remains after the citric acid treatment was analyzed by XRF (Table 5). The obtained result indicated that < 1% of CaO was dissolved.

**Table 5:** Chemical analysis by XRF of the precipitate obtained after PGF treatment with Na_2_CO_3_ followed by leaching with citric acid

Analyte	Compound formula	Conc. (%)
F	F	< 0.1
Na	Na_2_O	0.1
Mg	MgO	< 0.1
Al	Al_2_O_3_	< 0.1
Si	SiO_2_	1.8
P	P_2_O_5_	0.2
S	SO_3_	0.8
Ca	CaO	35.1
Sr	SrO	0.2
Ti	TiO_2_	< 0.1
Fe	Fe_2_O_3_	0.6
Ni	NiO	< 0.1

### REEs purification

**Fig. 4:**
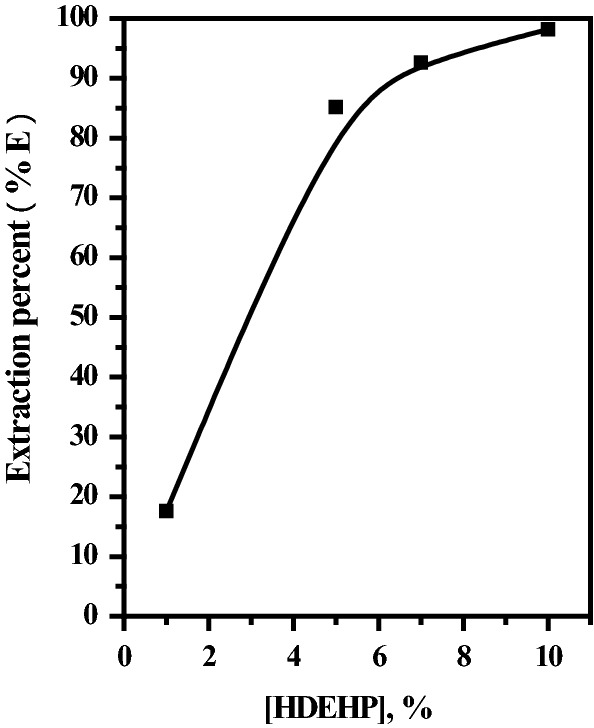
Effect of HDEHP concentration on the extraction of total REEs from simulated solution. T = 25 °C L:S ratio = 1:1 pH = 3.0

Based on the analysis of the precipitated obtained after leaching out of REEs with citric acid, Table 5, it is clear that some impurities such as Ca, Sr, Fe, etc. are present in the REE leaching with citric acid. Therefore, to purify the REE leach citrate solution from these impurities, solvent extraction was used for this purpose. In this respect, di-ethyl hexyl phosphoric acid (HDEHP, H_2_R_2_) is widely used in the extraction and purification of REEs present in different acidic media. [37] In this concern, a simulated solution containing REEs with the same ratios as present in the PGF sample was prepared in a citrate medium. Extraction of REEs with different concentrations of HDEHP in kerosene was carried out at an equilibrium time of 15.0 min. and 25 °C. The results obtained are presented graphically in Fig. 4, as a relation between % E and HDEHP concentration. The obtained results indicated that 10.0% is the proper concentration of HDEHP in kerosene for almost quantitative extraction of REEs from the citrate medium.

**Fig. 5:**
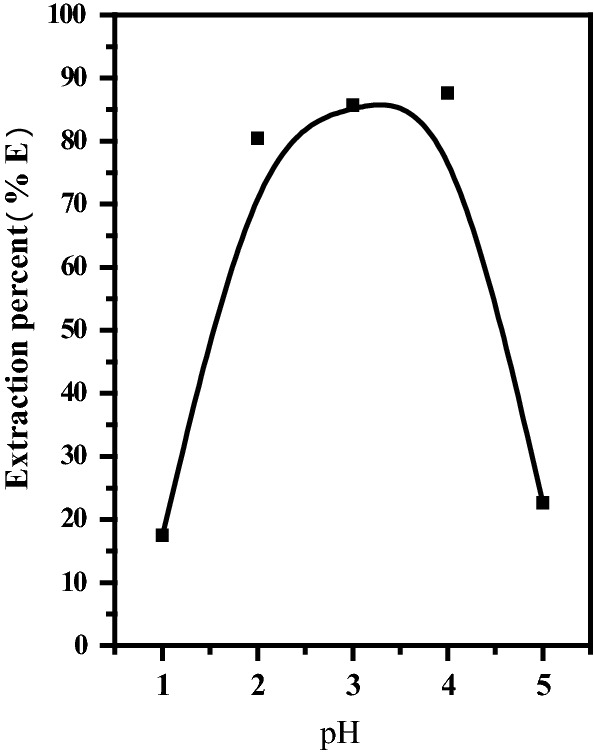
Effect of pH on the extraction of REEs from simulated solution. [HDEHP] = 5.0% contact time = 15.0 min

The pH results of the citrate acid concentration in the extraction process were given in Fig. 5 and found that the pH ranging from 3.0 to 4.0 is the most suitable for quantitative extraction of REEs. The extraction equilibration was as follows in Eq. (4): [38]

4 $${\text{REE}}^{ + 3} \,\left( {{\text{aq}}} \right)\, + \,3{\text{H}}_{2} {\text{R}}_{2} \,\left( {{\text{org}}} \right)\,{\text{REE}}\,\left( {{\text{HR}}_{2} } \right)_{3} \,\left( {{\text{org}}} \right) + \,3{\text{H}}^{ + } \,\left( {{\text{aq}}} \right)$$

The REEs leaching solution, obtained from the treatment of PGF with sodium carbonate and then with citric acid was contaminated with other elements, as previously mentioned. This solution was purified by extracting REEs with 10.0% HDEHP in kerosene at pH 3.0 for 15.0 min at 25 °C The extracted REEs were stripped by 0.5 mol/L H_2_SO_4_. The stripped solution was analyzed by ICP-OES to determine REE concentration, Table 6. Also, XRF analysis was carried out to determine the major impurities present in REEs. The obtained result is given in Table 7. Comparing this table with that of the original solution, Table 1, it is clear that the REEs produced were found to be free from fluoride and aluminum. The stripped solution contained no more than 0.4% calcium, whereas the unpurified REEs contained 35.9%. In addition, silica decreased from 9.95% to 0.3%. Other impurities are not more than 0.1%.

**Table 6:** Chemical analysis of REEs in the stripping solution by ICP–OES

Element	Concentration (mg/kg)
La	98.5
Ce	211.3
Pr	19.8
Nd	–
Sm	–
Er	67.3
Y	12.8
Yb	–
Total REEs	~ 409.7 mg/kg ~ 85.2%

**Table 7:** Chemical analysis of stripped solution by X- ray fluorescence (XRF)

Analyte	Compound formula	Conc. (%)
F	F	–
Na	Na_2_O	< 0.1
Mg	MgO	< 0.1
Al	Al_2_O_3_	–
Si	SiO_2_	0.3
P	P_2_O_5_	0.1
S	SO_3_	1.3
Ca	CaO	0.4
Sr	SrO	< 0.1
Ti	TiO_2_	< 0.1
Fe	Fe_2_O_3_	0.1
Ni	NiO	< 0.1

**Fig. 6:**
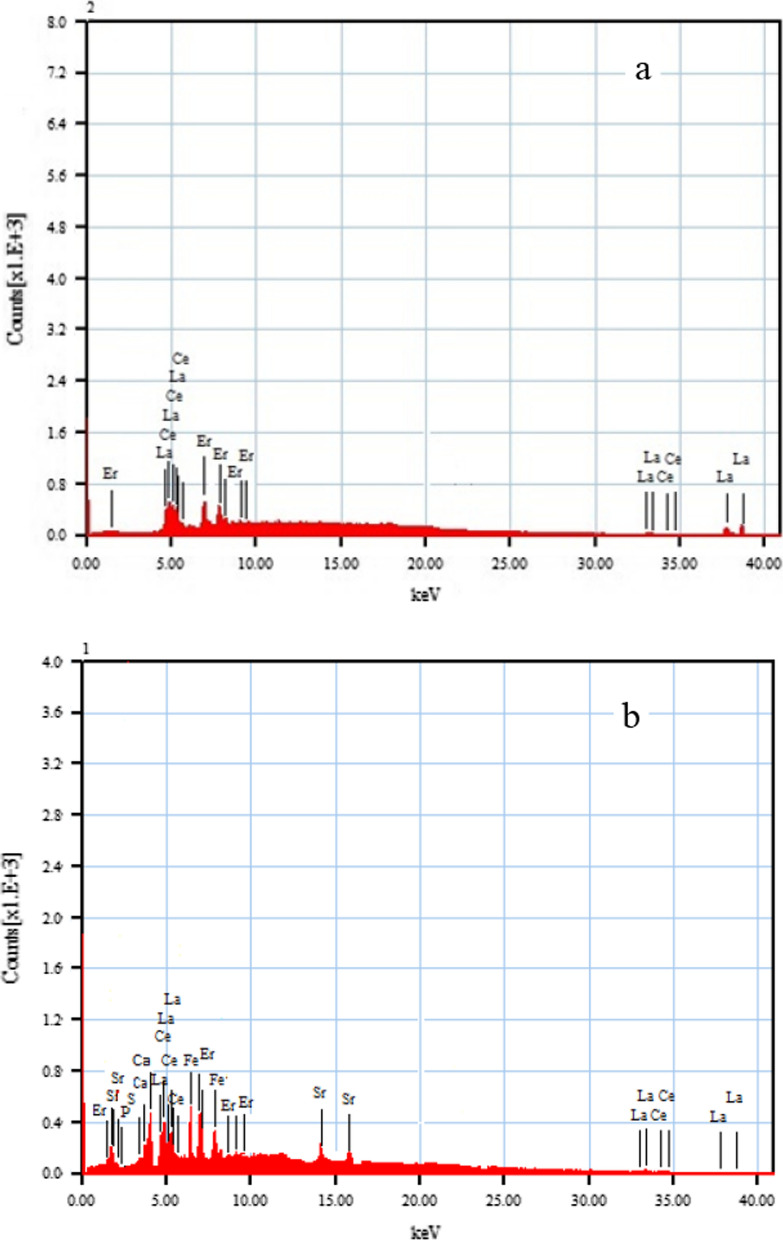
X- ray fluorescence analysis of precipitate by oxalic acid of **a** simulated solution, **b** stripped solution

The stripped and the simulated solutions were further treated with 10.0% oxalic acid to precipitate the REEs to be analyzed by XRF (Fig. 6a and b, respectively).

From Tables 6, 7 and Fig. 6, it is concluded that the developed procedure can recover REEs from PGF with an efficiency of 85% and a purity of 97.7%.

The summary of the main procedures developed was given in Table 8. The different leaching processes presented in the table indicate that a combined pre-treatment with alkali followed by one cycle with citric acid is so far the most efficient process for the REEs leaching from the PGF matrix.

**Table 8:** The summary of the main procedures developed

Leaching process	REEs concentration, mg/kg					% Total REEs	References
	Y	La	Ce	Pr	Er		
3 cycle 3.0 M nitric acid	11.9	85.9	159.2	10.7	49.3	66.0	[14]
3 cycle citric acid	8.3	95.6	180.2	17.7	59.3	83.4	[13]
Alkali treatment + 1 cycle nitric acid	8.8	95.8	206.6	19.5	70.7	75.1	This work
Alkali treatment + 1 cycle citric acid	13.9	100.7	215.3	20.9	69.4	87.4	This work

**Fig. 7:**
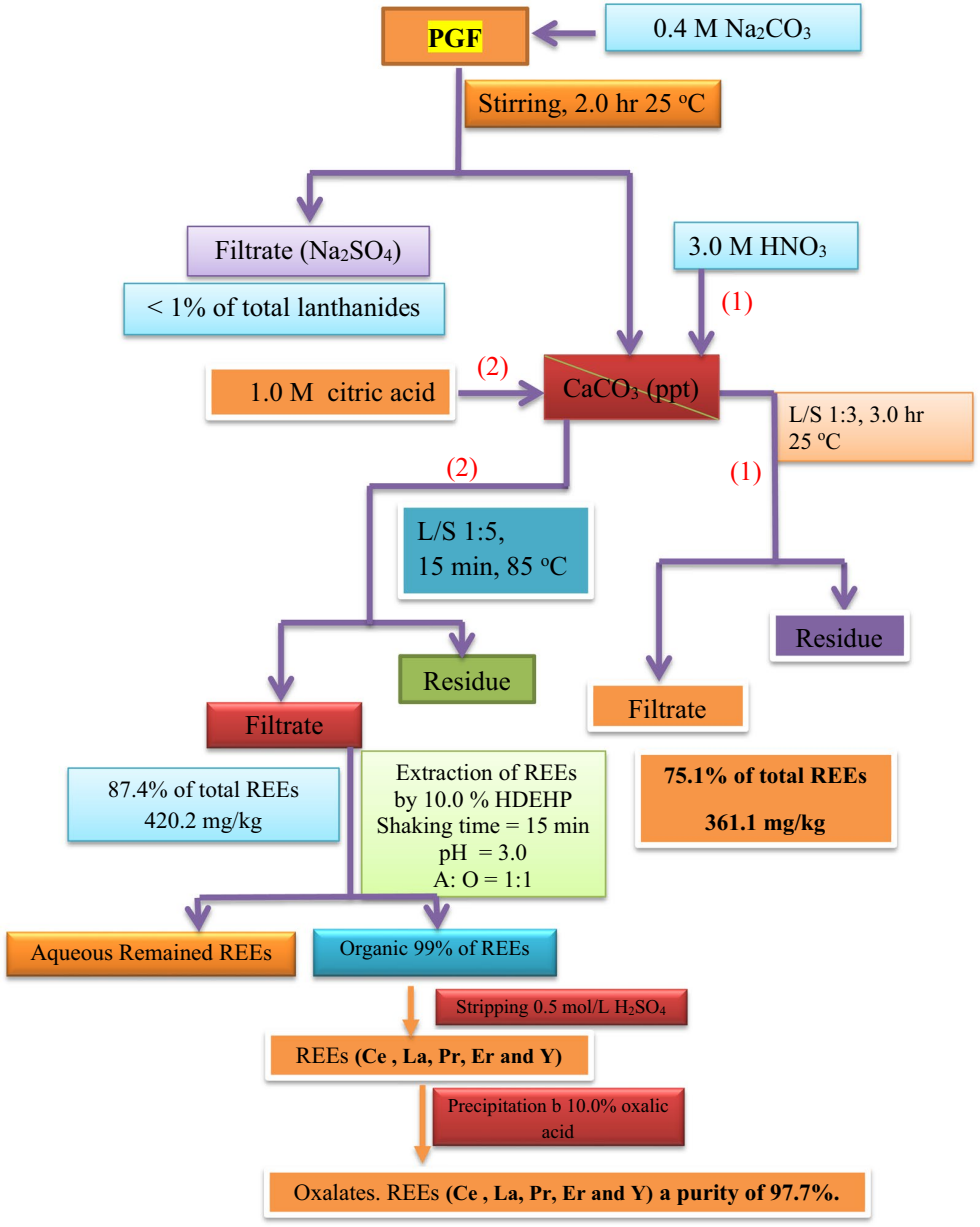
Flow diagram for REEs leaching from PGF with nitric acid or citric acid after carbonate precipitation

A proposed flow sheet for the process based on nitric acid as well as citric acid is given in Fig. 7.

## Conclusions

 The total REE content in PGF is about 481.0 mg/kg. The major components of the REEs are Ce, La, Er, Pr, and Y. Alkali treatment of PGF produces soluble sodium sulfate as a product and a precipitate of calcium carbonate containing REEs. REEs was recovered from CaCO_3_ by leaching with HNO_3_ acid or citric acid. Based on the obtained results, maximum leaching of 75.1%, 361.10 mg/kg of REEs from CaCO_3_ after one cycle leaching by 3.0 mol/L nitric acid at L/S = 3/1, agitation time of 180.0 min., and at a temperature of 25 °C. In this respect, La is the most leached element from PGF with an efficiency of more than 81.7%, followed by 76.9% for Ce, 75.0% for Er, 65.3% for Pr, and finally 37.9% for Y. While, the maximum leaching of 87.4%, 420.2 mg/kg of REEs from CaCO_3_ after one cycle leaching by 1.0 mol/L citric acid, L/S = 5/1, agitation time of 15.0 min., and 85 °C. The leaching efficiency of citric acid in the final leach solution followed the order; Ce (92.0%) > Er (87.7%) > La (86.1%) > Pr (77.1%) > Y (63.5%). Purification of REEs from citrate leach solution was carried out by 10% HDEHP in kerosene at pH 3.0 and shaking time of 0.25 h at room temperature. The extracted REEs were stripped by 0.5 H_2_SO_4_. This procedure can recover REEs from PGF with an efficiency of 85.0% and purity of 97.7%.

## Data Availability

All data generated or analyzed during this study are included in this article.
